# Organoids in cancer therapies: a comprehensive review

**DOI:** 10.3389/fbioe.2025.1607488

**Published:** 2025-07-22

**Authors:** Jiang Xin-Yi, Wang Yan-Ran, Di Pin-Ru, Qian Shi-Yi, Jiang Hai-Tao

**Affiliations:** ^1^ Department of General Surgery, Ningbo No. 2 Hospital, Ningbo, Zhejiang, China; ^2^ School of Clinical Medicine, Hangzhou Medical College, Hangzhou, Zhejiang, China

**Keywords:** organoids, cancer therapy, disease modeling, drug screening, personalized medicine

## Abstract

Organoid technology has significantly advanced biomedical research, offering deep insights into tumor biology and therapeutic efficacy. While existing publications have covered organoid applications, this review uniquely stresses their transformative role in cancer research. We highlight their importance in studying intratumoral heterogeneity and microenvironment interactions. Our analysis addresses knowledge gaps by detailing how organoids function as models in cancer initiation, drug screening, target identification, and sensitivity assessment. We also explore their applications in personalized medicine, such as developing patient-derived models for treatment prediction and immune therapy evaluation. This review discusses the latest progress in using organoids for cancer treatment, like predicting patient responses to precision medicine. However, challenges remain, including maintaining genetic stability and mimicking *in vivo* conditions. By addressing these limitations, this review provides a novel perspective on how organoid technology may overcome current barriers and drive innovation in cancer therapy. Our analysis suggests that advancements in organoid systems could enhance personalized treatment strategies and improve oncology patient outcomes.

## 1 Introduction

Cancer continues to represent a significant threat to life and a major risk factor for morbidity and mortality on a global scale (Global, 2016) in spite of the remarkable advancements in cancer research, diagnosis, detection and treatment made in recent decades. Currently, the most commonly used cancer models for research include human cancer cell lines, animal tumor tissue transplantation models and organoid models, which have been proposed in the last decade. This comprehensive review aims to synthesize recent breakthroughs in tumor organoid technology, evaluate its transformative role in overcoming the constraints of existing models, and delineate how organoids are reshaping precision medicine in cancer therapy.

An organoid is a combination of organ-specific cell types developed from stem cells or organ progenitor cells and organized in an organ-specific manner that emulates the process of *in vivo* cell sorting and spatial lineage qualification (Lancaster and Knoblich, 2014). This process exhibits three characteristics: (i) Specificity: The presence of specific cell types from different organs; (ii) Functionality: The ability to perform the functions of organs, including contraction, secretion, filtration, and so on; and (iii) Spatiality: Immortalized cancer cell lines, while widely accessible, undergo genetic drift during long-term culture, losing the heterogeneity and microenvironmental context of original tumors (Wilding and Bodmer, 2014). This compromises their predictive value for clinical drug responses. In comparison to human cancer cell lines, organoids exhibit greater stability, less prone to undergo genetic alterations and heterogeneity during cell proliferation. Studies have demonstrated that following the sequencing of cancer-related genes in colon tumor organoid models and their primitive tumors, there is a 90% similarity between the organoid models and biopsies in terms of somatic mutations and DNA copy number (van de Wetering et al., 2015). This confirms that organoids are capable of maintaining. Animal xenograft models suffer from interspecies differences in tumor-stroma interactions, immune responses, and drug metabolism. Their high cost, prolonged timelines, and ethical constraints further limit scalability for high-throughput studies (Hidalgo et al., 2014). In comparison to animal models, organoids offer a more cost-effective, time-efficient, and genetically stable alternative (Tuveson and Clevers, 2019). Despite their inability to restore immune system interactions during natural proliferation, they provide a valuable research tool for studying cancer biology.

The cultivation of tumor-derived organoids from tissue- or tumor-specific stem cells is dependent on the advancement of three-dimensional (3D) culture technology. One of the earliest known tumor-derived organoids was developed by Sato et al. The culture environment was adapted to achieve unlimited proliferation and spatial arrangement of tumor cells based on the culture conditions of mouse colonic crypts. This was achieved by adding Wnt3A, nicotinamide, as well as Alk small molecule inhibitors and p38 inhibitors (Sato et al., 2011). To date, successful tumor organoid models have been cultured, including those derived from colon carcinoma (Barbáchano et al., 2021), Prostate carcinoma (Mai et al., 2021), gastric carcinoma (Huo et al., 2023), Renal carcinoma (Batchelder et al., 2015) and Head and Neck squamous cell carcinoma (Qi et al., 2023). Based on the simulation of pathological mechanisms of tumor development and mutations in different signaling pathways, tumor organoids have demonstrated considerable potential in anti-cancer drug screening and personalized medicine. Despite the nascent state of organoid applications in guiding clinical treatment, there is a growing demand for the use of tumor organoids to inform patient treatment.

## 2 Organoids in cancer modeling

### 2.1 Recapitulation of intratumor heterogeneity and microenvironment in organoids

Millions of cancer cells and their embedded tumor-associated microenvironments collectively constitute a tumor, which exhibits heterogeneity that can influence cancer progression and create some resistance to clinical therapy. Taken together, intratumor heterogeneity is the result of a complex, multifactorial-driven integration of genetic genomic, epimolecular, and microenvironmental changes (Marusyk et al., 2020).

At present, there is no clear conclusion on the molecular mechanism of tumorigenesis, and we hope to elucidate its heterogeneous developmental progression with the help of clinical models and seek new treatment options. For example, Mo et al. (2022) demonstrated that organoids are able to capture tumor heterogeneity between patients and individuals by constructing a biobank of organoids from primary colorectal cancer with liver metastasis and analyzing them comprehensively at the multi-omics level. As another example, Jacob et al. (2020) constructed patient-derived glioblastoma-like organoids (GBOs) and confirmed the ability of the organoids to retain inter-versus intratumor transcriptomic and genomic heterogeneity to a high degree by comparing the cell types and molecular features of the parent tumors. In conclusion, living organoid biobanks constructed using patient-derived tumor cells can provide powerful credentials and abundant resources for studying the molecular heterogeneity and biological behavioral characteristics of cancer at the genetic level.

Tumor development is not only subject to the transformative and regulatory effects of genetic and molecular phenotypes of normal cells, but also requires the infiltration of a highly abnormal microenvironment (Marusyk et al., 2020).

Among them, tumor-associated microenvironment (TME) consists of vascular tissues and lymphatic networks, fibroblasts, various immune cell subpopulations, extracellular matrix, and various signaling factors in and around the tumor (Wang et al., 2022b), which plays a crucial role in sustaining the cancer process. Therefore, the use of organoid technology to mimic the corresponding tumor microenvironment to construct “immune-tumor organoids” has a good application prospect to explore the development of cancer.

However, as of now, traditional organoid models are unable to replicate the stromal components of the parental cells including the various immune cell subpopulations therein (Drost and Clevers, 2018), and there is an urgent need to introduce the cellular components of TME environments in order to better mimic the corresponding microenvironmental dynamics systems. Several co-culture strategies are available today in an attempt to mimic the corresponding tumor microenvironment, with direct co-culture, microfluidic 3D culture, and ALI culture being the three main methods. These methods each have their own advantages and disadvantages (Li Y. et al., 2024) (Table 1).

**TABLE 1:** Comparison of the proposed microenvironmental co-culture methods for the three organ types.

Features	The air-liquid interface (ALI)	Submerged substrate gel culture (direct)	Microfluidic 3D culture
Training environment	Transwell (divided into upper and lower chambers)	Containing substrate gel culture plate	3D microfluidic device
Advantages	Maintaining the genetic characteristics and molecular phenotype of parental tumor tissue is more supportive of long-term culture or passage culture	Simulate the circulatory system within the organization to provide a constant growth environment for organoids The miniaturization and integration of the system have improved the uniformity and controllability of organoids	Maintain the pathological and histological characteristics of parental tumors Better direct reconstruction and simulation of the corresponding tumor microenvironment
Disadvantages	The original system can only enrich tumor epithelial cells, and matrix cell components are easily lost Exogenous addition of immune cells is required to simulate the tumor microenvironment	Easily affected by limitations in equipment, technology, and other conditions Exogenous addition of immune cells is required to simulate the tumor microenvironment	Some original cellular components, such as immune cells,may be consumed during the culture process and may not be able to sustain long-term cultivation
Main applications	It mainly generates epithelial cell line like organs. At present, it has successfully established brain, stomach, esophagus, lung, liver, pancreas, kidney, kidney, salivary gland, breast cancer and other organ like models. Provided an effective platform for studying the interaction between tumor cells and immune cells	Mainly used for the development of immune drugs and screening of high-throughput sensitive drugs in personalized therapy	Mainly used to simulate the immune microenvironment and explore its specific mechanisms in tumor occurrence and development

The direct Submerged substrate gel culture represents a cornerstone technique in organoid research, with its origins tracing back to the seminal work by Clevers’ team on Lgr5+ intestinal stem cells (Sato et al., 2009). This methodology involves enzymatic or mechanical dissociation of fresh tumor tissues into single-cell suspensions, followed by embedding within laminin-rich basement membrane extract (Cultrex Stem Cell Qualified Basement Membrane Extract, BME). The embedded constructs are subsequently immersed in tissue-specific media supplemented with defined growth factors or pathway inhibitors. Notably, epithelial organoid growth typically necessitates Wnt agonists, receptor tyrosine kinase ligands, BMP inhibitors, and TGF-β antagonists.

However, traditional Matrigel-embedding systems rely predominantly on passive diffusion for nutrient/waste exchange,a process severely constrained by increasing organoid size (Liu et al., 2024). To address this, microfluidic 3D culture platforms have emerged. These systems employ microfluidic chips featuring a central gel chamber flanked by bilateral perfusion channels. Operationally, cell-Matrigel mixtures are injected into the central chamber, while culture media are perfused through adjacent channels (Jenkins et al., 2018). This design enables microscale modeling and functional integration through high-density tumor cell seeding within microporous architectures.

Both Matrigel-embedding and microfluidic approaches require exogenous immune cell supplementation to reconstruct the tumor microenvironment (TME). In contrast,the air-liquid interface (ALI) culture establishes a biphasic system using Transwell inserts. Tumor fragments embedded in collagen matrices are exposed to air in the upper chamber (gas phase), while basal nutrients diffuse upward through microporous membranes from serum-supplemented media below (liquid phase) (Mu et al., 2023).

Critically, ALI’s non-enzymatic processing and biphasic design optimally preserve native immune components, positioning it as the gold standard for *in situ* TME modeling.

As Dijkstra et al. (2018) have established an organoid model of a tumor-like microenvironment, which is a co-culture system containing IL-2 mediators using anti-CD28 and anti-PD-1(Programmed Death-1,PD-1) antibodies against peripheral blood mononuclear cells (Peripheral blood mononuclear cells (PBMC) were stimulated with anti-CD28 and anti-PD-1(Programmed Death-1,PD-1) antibodies to induce the generation of lymphocytes, which were then co-cultured with interferon-γ (IFN-γ) pretreated monocyte suspensions. In the process of exploring the optimal solution of the co-culture method, the researchers found that the components contained in the medium could theoretically penetrate the stromal gel and mimic the biological processes in the patient’s body, but in reality, it is difficult for the immune cells, which are the main components, to penetrate the stromal gel and interact with the tumor. Although the ALI method can directly reconstruct the natural stroma and immune environment, which more realistically resembles the internal environment of the human body, the immune components are depleted over time, and it is still unable to provide a stable survival environment for organoids for a long period of time (Li Y. et al., 2024).

In fact, in addition to the immune cell component, the vascular component and the fibroblast component are also indispensable components of cancer organoid mimicking the tumor microenvironment (Li Y. et al., 2024). As far as the current organoid models are concerned, the experiments are still insufficient to make up for the above three components, such as the maintenance of the culture environment is not stable for a long period of time, and the co-culture conditions still need to be optimized, which is still a major challenge for the development of “immune-tumor” organoid. For the currently established organoid model, it is still insufficient to compensate for the above three components in the experiments, such as the maintenance of the culture environment is not stable for a long period of time, the cost of the culture cycle is too high, and the co-culture conditions still need to be optimized, which is still a major test for the development of “immune-tumor” organoid.

### 2.2 Use of organoids in studying cancer initiation, progression, and metastasis

Cancer formation, development, deterioration and metastasis is an interrelated multifactorial and gradual process, and the establishment of the corresponding tumor organoid biobank model can help us to understand the specific mechanism of cancer at different stages of evolution, which is of great significance for clinical screening and diagnosis, prevention and treatment.

On the one hand, some infectious agents have been recognized as important risk factors for cancer, such as gastric cancer and *Helicobacter pylori*, gallbladder cancer and *Salmonella enterica*, liver cancer and hepatitis viruses, as well as nasopharyngeal cancer, gastric cancer and lymphoma and EBV. However, the exact mechanism of association as well as the causal relationship is not yet clear, and we would like to explore the exact process with the help of organoid-pathogen binding co-culture (Xu et al., 2018). For example, Buti et al. (2020), by constructing a co-culture system of *H. pylori* with gastric organs, found that its virulence factor, CagA, bound to the Apoptosis stimulating protein of p53-2 (ASPP2) to form a complex ASPP2 induces the remodeling of cell polarity complexes, resulting in the loss of cell polarity and the promotion of epithelial-mesenchymal cell transformation, which affects the development and metastasis of gastric cancer. On the other hand, the continuous accumulation of mutations as an important genetic basis for cancer development implies that it is crucial to clarify the original mutational spectrum of tumorigenesis (Davies et al., 2017). Although tumorigenesis is the end result caused by the continuous accumulation of genetic mutations, only a few of these mutations lead to cancer development (Stratton et al., 2009), and we call such mutations as driver mutations. Tumor metastasis, on the other hand, is considered to be the end result of an invasive biological process of cells with a cascading, multistep nature, which occurs as a result of a combination of genetic or phenotypic inheritance within tumor cells and non-tumor stromal cells driven by a combination of genetic or phenotypic inheritance and non-tumor stromal cells (Valastyan and Weinberg, 2011). For example, Drost et al. (2017) used CRISPR-Cas9 technology to knock down DNA repair genes that play a key role in human colonic organoids, and accurately mimicked the spectrum of mutations in mismatch repair-deficient colorectal cancers by delayed subcloning and whole genome sequencing. In this study, it was also found that mutation accumulation in mismatch repair gene MLH1-deficient quasi-organs was driven by replication errors. This shows that tumor-like organs can assist us in better understanding the mechanisms of cancer development and metastasis at the genetic level.

In addition, circulating tumor cells (CTCs), a population of tumor cells that cause metastasis after being shed from the site of tumor primary focus and entering the peripheral blood circulation system, are usually used as an effective key tool for clinical cancer diagnosis and prognosis (Deng et al., 2022). However, CTCs are actually low in peripheral blood and are difficult to be captured for further monitoring and examination in clinical trials. Gao et al. (2014) human successfully cultured and established a prostate tumor organoid model by using by using circulating tumor cell biopsy technology in conjunction with a 3D organoid system. In addition, the organoid in this study systematically summarized the molecular diversity of prostate cancer subtypes and pointed out the targets of multiple gene mutations. This suggests that even CTCs with sparse cell numbers can be modeled by organoid technology, which is potentially valuable for exploring the clinical development of cancer, early diagnosis and screening, and prognostic and therapeutic monitoring. Tumor stem cells, also known as tumorigenic cells, are a subpopulation of cancer cells with the potential for self-renewal and multidirectional differentiation. Their tumorigenicity, which confers tumor metastability, susceptibility to recurrence, and chemoresistance, is a potentially important target for clinical cancer treatment (Fan et al., 2023).

### 2.3 Organoids as models for studying cancer stem cells and tumor evolution

Tumor-derived sphere culture method is currently one of the more applied and representative three-dimensional culture methods in tumor stem cell research, which mainly utilizes primary tumor cells with stem cell-like characteristics to expand *in vitro* in the form of floating spheres. The popular cancer stem cell (CSC) model suggests that the self-renewal and differentiation ability of cancer stem cells can occur to form complete tumor structures. Wang et al. (2023) found that c-Myc, CD44, CD133, ALDH1A1 were positively correlated with breast cancer phenotype by analyzing the transcriptome spectrum of 1,533 breast cancer patients. Jun et al. (2021) demonstrated through their research that TIPRL, LC3, CD133, and CD44 are potential biomarkers for early liver cancer. Thirusangu et al. (2022) found that PFKFB3 is a new target to regulate CSC and its related therapeutic drug resistance markers YAP/TAZ and ABCG2 in the small cell liver cancer model. Wang et al. (2023), Jun et al. (2021), Thirusangu et al. (2022) although biomarkers have been applied to CSC in different cancer models, the identification of CSC in solid tumors is still controversial (Ishiguro et al., 2017). Organoid technology, as an emerging research tool, will become an important research platform to promote the value-added differentiation of tumor stem cells and the mechanism of cancer development by utilizing cell mass constructs formed by cells with stem cell potential after *in vitro* 3-dimensional culture (Xu et al., 2018).

At the same time, we still have not clarified the dynamic process of tumorigenesis and evolution. For example, even after the removal of patient-derived tumor cells from the body and their application to laboratory research, the process of tumor growth and development is only limited to the final stage of cancer evolution, and it is not possible to elucidate the process of tumor growth and development from a whole timeline (Graham and Sottoriva, 2016). However, if the application of organoid technology is combined with genome sequencing, it will open up a completely new way of thinking for exploring the specific mechanisms of tumor development. The organoid lineage biobank of human bladder cancer constructed by Lee et al. (2018) not only summarizes the pathohistological and molecular biological features of human bladder cancer, but also reveals the lineage of this type of bladder cancer through the systematic analysis of exome sequencing. Revealed the linear and branching tumor evolutionary patterns of this organoid. Furthermore, for example, Zhao et al. explored the heterogeneity and evolutionary evolution of hepatobiliary tumors by establishing seven hepatobiliary tumor organoids, and found that HCC272 with high epithelial-mesenchymal transition status has broad-spectrum drug resistance by using single-cell RNA technology. Norkin et al. (2021) has developed a targeted organoid sequencing (TORNADO-seq) platform to monitor colorectal cancer drug discovery through targeted RNA-seq. In addition, single-cell RNA-seq and cancer organoid models have shown good advantages in studying the heterogeneity of primary tumor microenvironment and promoting chemotherapy regimen selection (Chen et al., 2024).

**FIGURE 1:**
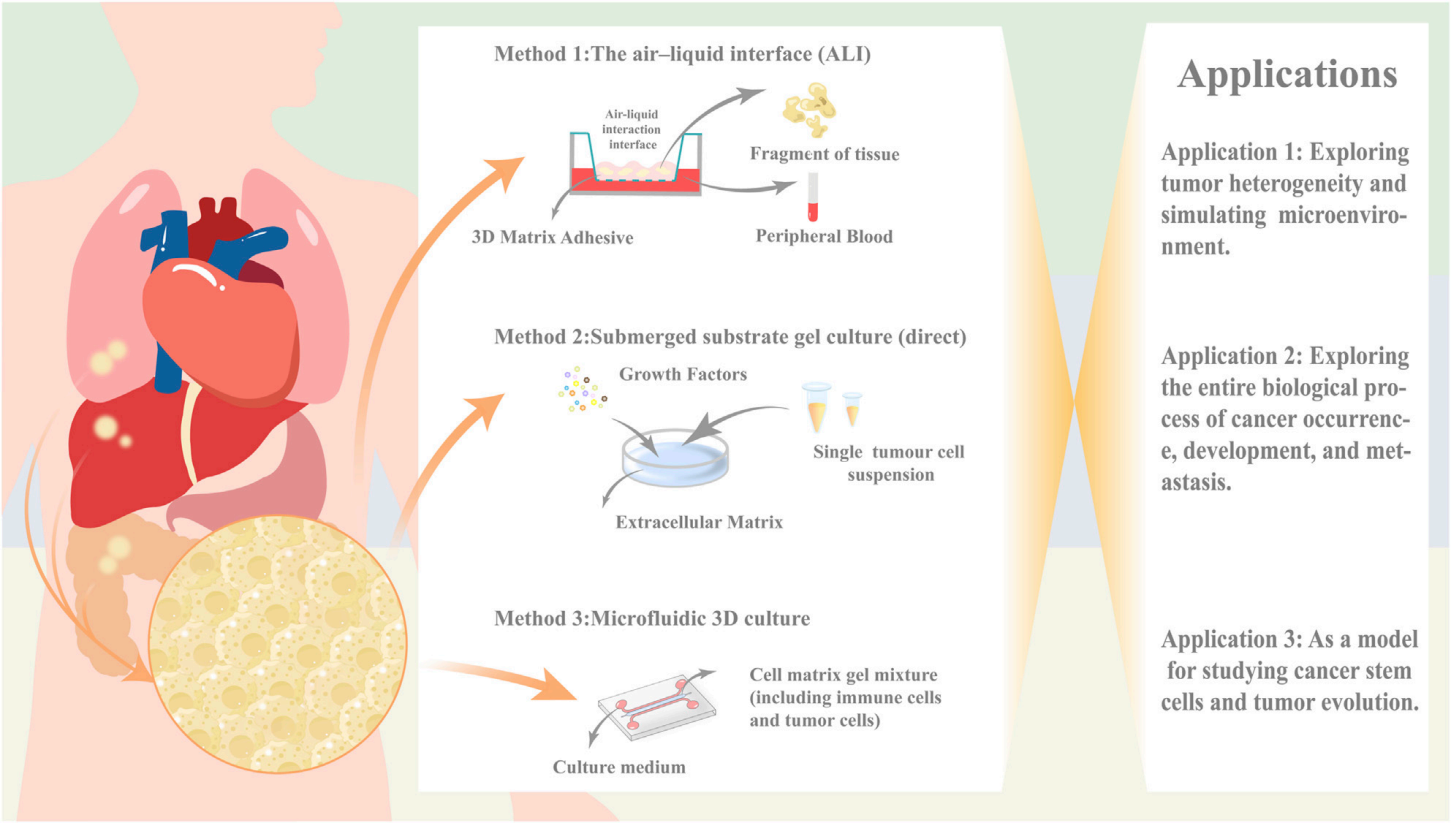
Organoid Culture Methods and Their Applications in Cancer Research.

In summary, organoid models have shown promising application prospects in simulating tumor heterogeneity and tracking cancer stem cells, which is conducive to promoting drug resistance screening and precision medicine (Figure 1).

## 3 Organoids in drug discovery and development

### 3.1 High-throughput drug screening using organoids

Organoid models have attracted attention for their high-throughput screening capabilities, allowing for efficient compound screening, especially in the field of cancer drug discovery research, where they have become a research hotspot. Han et al. (2021) demonstrated that small molecule drugs such as imatinib and mycophenolic acid can significantly inhibit the entry of severe acute respiratory syndrome coronavirus 2 into cells by establishing lung and colon organoid models, validating the use of organoid models for screening candidate drugs for patients with novel coronavirus infections. Bergdorf et al. (2020) dissociated organoids into single cells and plated them in 384-well format for high-throughput drug screening, showing that organoid cells treated with TrypLE exhibited higher survival rates and better functional states, with significant effects on thyroid and melanoma organoid lines. Choo et al. (2021) developed a novel quantitative high-throughput imaging analysis by constructing organoids from cultured tissues derived from patient-derived xenografts (PDX), confirming that organoids with different phenotypes from prostate cancer can be used to test drug treatments or screen compound libraries at wide dose responses, and carefully quantifying growth and morphological changes at the well level and per organoid. Despite the advantages organoid models exhibit in clinical drug screening, providing significant reference value for drug research, there are still certain limitations, such as low purity of organoid models, difficulty in construction, and limited availability. Further research is needed to prove the potential of organoids in high-throughput drug screening and to enhance their reliability and application value.

### 3.2 Organoids in the identification of drug targets and biomarkers

Organoids can display *in vivo* gene expression and protein function and predict the progression of disease development, facilitating gene editing technologies. By establishing disease models, it deeply explores how the intervention of specific genes affects the development process of cancer and the efficacy of drugs, aiding in the discovery of new clinical treatment targets (Yujia et al., 2024). Norkin et al. (2021) and others have developed a rapid, highly reproducible targeted organoid sequencing (TORNADO-seq) that can be used to identify small molecule drugs capable of inducing differentiation in wild-type and cancer cells of the intestine. Taverna et al. (2020) constructed patient tumor-derived short-term lung adenocarcinoma organoids and found that tyrosine protein kinase receptor (Anexelekto, AXL) inhibitors can target and suppress SMAD4/TGFβ signaling and induce JAK1-STAT3 signaling, further confirming the effectiveness of organoids in identifying drug targets and providing feasibility for clinical cancer treatment. Huang (2022) and others have demonstrated through research that micro vascularized intestinal organoid chips have potential advantages in drug target screening and discovered that olfm4 can reduce cell inflammation caused by HR by inhibiting the NF-kappa B signaling pathway, thus designing targeted drugs to prevent and treat intestinal ischemia-reperfusion (I/R) injury. Therefore, organoid technology exhibits unique advantages in identifying drug targets and biomarkers, providing more precise and efficient tools for drug development and disease research, and is expected to play a more important role in future medical research and clinical practice.

### 3.3 Organoids in the study of drug sensitivity and the development of combination therapies

**FIGURE 2:**
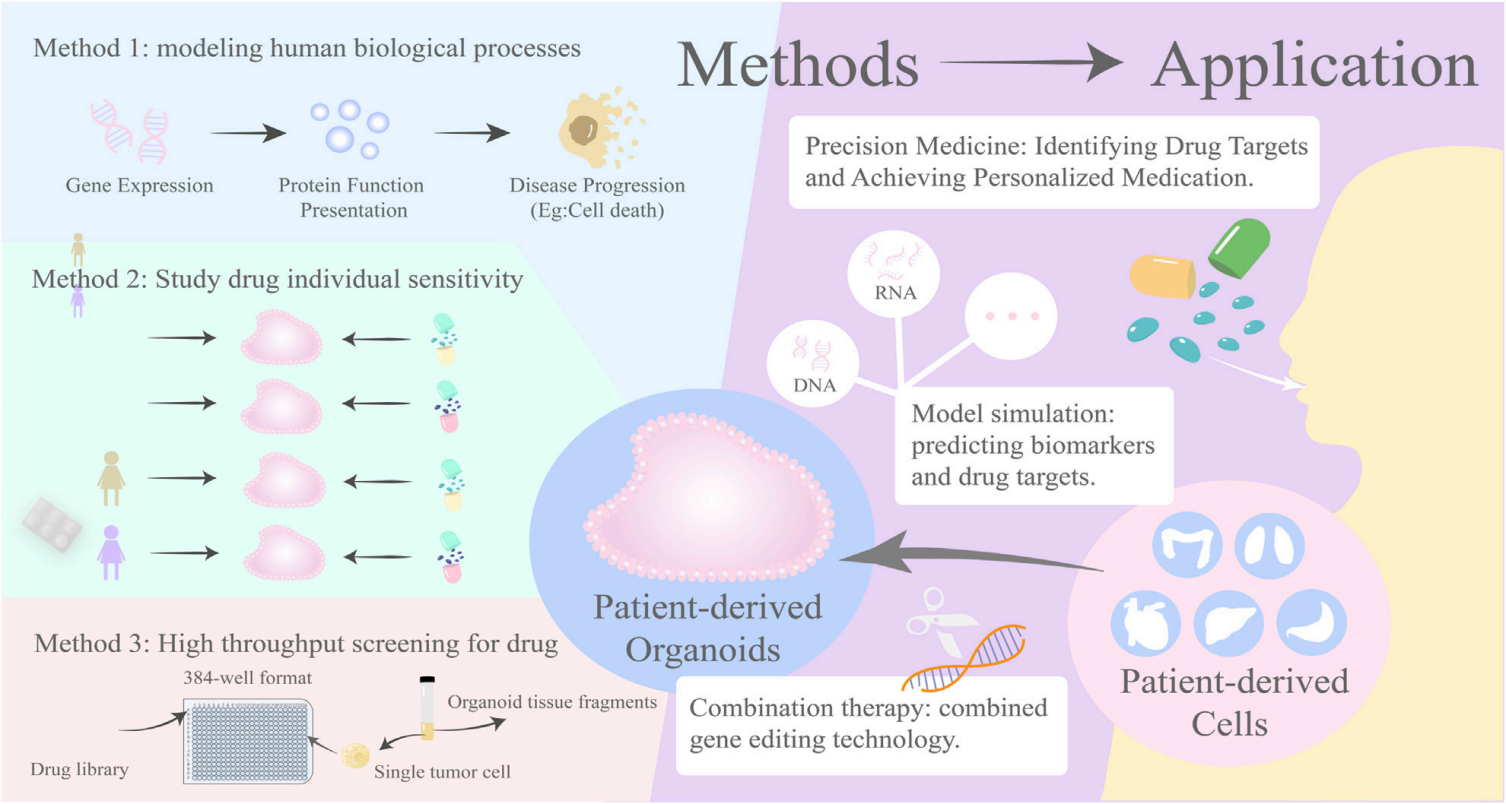
Organoids in the study of drug sensitivity and the development of combination therapies.

Organoids, as individualized clinical models, can provide personalized medication guidance through drug sensitivity testing, to improve treatment response rates and reduce chemotherapy toxicity (Peng and Zhu, 2023). Fan et al. (2025) conducted a drug sensitivity detection experiment on gastric cancer organoids and found that organoids cultured from different patient-derived gastric cancer tissues had different sensitivities to the same drug, and organoids cultured from the same patient-derived gastric cancer tissues had different sensitivities to different drugs. Wang et al. (2024) selected fluorouracil, gemcitabine, capecitabine, SN38, cisplatin, and oxaliplatin for single-drug sensitivity testing and also found similar results. In addition, organoids have shown significant results in combination therapy, including combination drug therapy and gene editing. Yan et al. (2022) compared the effects of single DCLK1 inhibitor and combined with EGFR inhibitor in a constructed TKI-resistant organoid model, and the results showed that inhibiting DCLK1 activity could restore the sensitivity of TKI-resistant tumor organoids, and DCLK1 inhibitor and EGFR inhibitor have a synergistic effect in inhibiting tumor growth. NBs are virus-like particles (VLP) derived from the mouse leukemia virus (MLV), and Tiroill et al. (2023) knocked out eGFP in mouse colon organoids using NBs to test their gene editing ability, proving that the combination of organoids and gene editing technology has lower toxicity and higher accuracy in treatment. Overall, drug sensitivity treatment of organoids is beneficial for overcoming patient resistance, enhancing drug efficacy, and providing reference indicators for patients to develop personalized combination medication, with the hope of achieving breakthrough progress in the field of cancer treatment in the future (Figure 2).

## 4 Organoids in personalized medicine

The US National Human Genome Research Institute defines personalized medicine as “an emerging practice of medicine that uses an individual’s genetic profile to guide decisions made in regard to the prevention, diagnosis, and treatment of disease. Knowledge of a patient’s genetic profile can help doctors select the proper medication or therapy and administer it using the proper dose or regimen” (Delpierre and Lefèvre, 2023). It aims to provide patients with precise diagnostic, therapeutic and preventive strategies based on individual genetic, environmental and lifestyle factors. With the continuous development of biotechnology, organoid technology is gradually becoming an important research hotspot for personalized medicine (Zhao et al., 2022). It can simulate the structure and function of organs in the body *in vitro*, providing a unique platform for in-depth study of disease mechanisms, drug response prediction and so on.

### 4.1 Use of patient-derived organoids for personalized treatment strategies

In recent years, patient-derived organoids (PDOs) have emerged as a promising model for the study of cancer. PDOs maintain the complex cellular composition and genetic diversity found in specific tumors, making them more representative of a patient’s individual disease characteristics (Abbasian et al., 2024). PDOs maintain the complex cellular composition and genetic diversity found in specific tumors, making them more representative of individual patient disease characteristics. The establishment of patient-derived organoids usually begins with the acquisition of tissue samples from patients, such as embryonic stem cells (ESCs), induced PSCs, and stem cells such as adult stem cells (ASCs), because of their versatile differentiation and self-renewal capabilities (Kim et al., 2020). Tumor-like organs can also be created from fresh tumor tissue using preoperative biopsies or postoperative tumor resection (Drost and Clevers, 2018). Tumor organoids can also be created from fresh tumor tissue using preoperative biopsies or postoperative tumor resection. Tumor stem or progenitor cells are isolated from samples taken from tumor fluid biopsies or by pasteurized brushing, and these cells are then placed in a culture environment containing a specific extracellular matrix (e.g., Matrigel), with the addition of appropriate growth factors (Borrell, 2010), while a suitable combination of growth factors, such as epidermal growth factor (EGF) and fibroblast growth factor (FGF), is added to promote organoid formation and growth. During the culture process, the culture conditions, including temperature, carbon dioxide concentration, nutrient supply, etc., need to be strictly controlled. After a period of time, the organoids gradually form and have a similar tissue structure and cellular composition as the source tissues, and are ready for subsequent analysis and application (Thorel et al., 2024).

### 4.2 Organoids in predicting patient response to chemotherapy and targeted therapies

Several studies have utilized tumor-like organs for chemotherapeutic drug sensitivity testing and prediction of response to targeted therapy. In colorectal cancer research, Chen et al. (2022) constructed two patient-derived colorectal tumor organoid (PDO) strains and investigated the sensitivity of colorectal tumor organoids to 5-fluorouracil (5-FU) and oxaliplatin, identifying potential therapeutic targets for the treatment of patients with oxaliplatin-resistant colorectal cancer. In addition, the study also uncovered several putative resistance genes and transcription factors (Vlachogiannis et al., 2018). In a study conducted by Vlach Giannis et al., a collection of *in vivo* biospecimen bank samples obtained from patients with metastatic and previously treated colorectal and gastroesophageal cancers showed remarkable similarity to the original patient tumors at the level of phenotypic and genotypic analysis. This similarity was further demonstrated by the ability to accurately predict patient response to targeted drugs or chemotherapy with 100% sensitivity and 100% negative predictive value. In lung cancer treatment, Ki et al. (2019) constructed a biobank of 80 organoids that showed responsiveness to targeted drugs such as olaparib, erlotinib, and crizotinib based on their genomic alteration status. This implies that the LC organoid biobank may be an effective tool for predicting therapeutic responses to patient-specific drug trials *in vitro*.

### 4.3 Organoids in the development of precision medicine approaches

**FIGURE 3:**
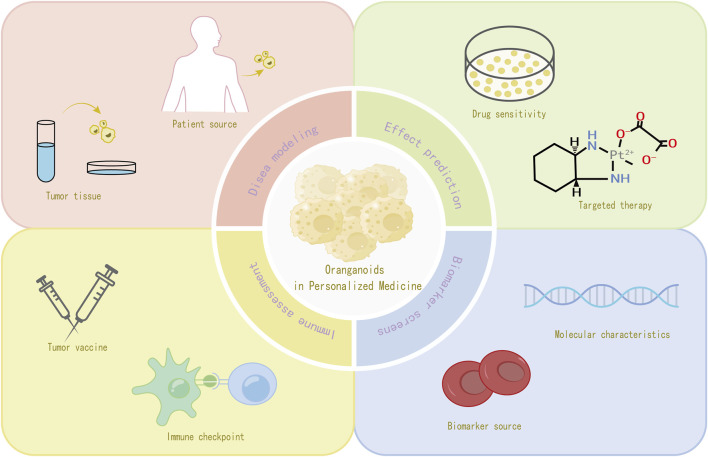
Organoids in the development of precision medicine approaches.

Precision medicine requires the identification of biomarkers to screen patients for benefit, and current assessments of key target expression or genetic abnormalities, while diagnostic, prognostic, and predictive, have limitations, such as lack of selectivity in molecular characterization and limited interpretation (Mirza et al., 2016). In contrast, organoid testing in precision medicine can determine the correlation between response to *ex vivo* therapy and the presence of predictive biomarkers of different types (e.g., DNA, messenger RNA, noncoding RNA, and proteins, as well as other biomarkers) and sources (e.g., tumor, blood, and urine, as well as other sources) (Thorel et al., 2024). For example, increased c-Jun phosphorylation after treatment exposure was observed in cisplatin-sensitive gastric cancer organoid therapy (Tsukamoto et al., 2022); in pancreatic cancer organoid studies, a number of gene expression signatures associated with drug resistance were identified by comparing transcriptomic data from chemotherapy-sensitive and drug-resistant organoids (Li et al., 2023). These features can be used as potential biomarkers to assist clinicians in developing personalized treatment plans and improving the precision of treatment (Figure 3).

### 4.4 Organoids in the evaluation of immunotherapeutic responses

Tumor-like organs can be used to predict patient response to immune checkpoint inhibitors (Thorel et al., 2024). The broad approach is to expose patient-derived tumor-like organs to immune checkpoint inhibitors and observe changes in the growth of the organs, immune cell infiltration, and alterations in the expression of relevant immune molecules (Scognamiglio et al., 2019). For example, co-culture of an organoid model of glioblastoma with chimeric antigen receptor-T (CAR-T) cells demonstrated antigen recognition, subsequent T-cell activation, and tumor cell death, thus highlighting the potential of organoids for testing antigen-specific CAR-T cell therapy responses (Jacob et al., 2020).

In addition to immune checkpoint inhibitors, Tumor Organoids can be used as a testing platform for vaccine immunogenicity and efficacy in tumor vaccine research (Wang et al., 2022a). By incubating tumor organoids with the vaccine and observing the changes in the expression of antigen-presenting molecules on the surface of the organoids, as well as the recognition and killing activity of immune cells against the organoids, we can determine whether the vaccine can effectively stimulate the immune system to generate an immune response against tumor cells. In recent years, some studies (Demmers et al., 2020) have correlated organoid proteomics with HLA peptide ligandomics, it was found that tumor-specific ligands derived from DNA damage and tumor suppressor proteins were significantly present in tumor cells, and there was heterogeneity in HLA peptide expression among individual patients, which led to the speculation that potential peptide tumor vaccines might be a feasible way to reduce the risk of immune escape.

## 5 Discussion

At the present time, the prevention, control and treatment of malignant tumors are in a critical situation. There are several major directions in cancer treatment, including the improvement of early diagnosis rates, the development of new strategies for individualized treatment and the prolongation of the life cycle of patients. Organoids, an emerging tissue model that mimic the development of cancer *in vivo*, have demonstrated considerable potential for cancer therapy.

As previously stated above, organoids have shorter culture cycles, lower consumption costs, and stable genetic heterogeneity compared with traditional cellular and animal models. Additionally, researchers can simulate the microenvironment of tumors *in vivo* by regulating the composition of the culture medium, thereby more accurately reproducing the *in vivo* process of tumor development. This contributes to the development of tumor stem cell culture and provides a foundation for subsequent clinical therapeutic research. Nevertheless, organoid culture remains hindered by two significant challenges. The first issue is the lack of reproducibility. Velasco et al. demonstrated that the reproducibility of organoid culture can vary under identical conditions (Velasco et al., 2019). It has been postulated that this may be attributable to the utilization of disparate batches of serum during the construction of the models. In particular, foetal bovine serum (FBS) derived from the blood of foetal cows comprises a multitude of peptides, proteins, lipids, hormones, carbohydrates and small molecule nutrients (LeSavage et al., 2022). The harvesting of serum is subject to seasonal and geographic variations, which result in discrepancies in the concentration of soluble components between suppliers and batches. In order to achieve consistency, synthetic materials such as HA, PEG and gelatin have been widely developed as substrates for organoid cultures (Zhang et al., 2023). Consequently, future research must identify the factors that contribute to the generation of reproducible organoids. Otherwise, such variability limits the reproducibility of organoid cultures on a large scale and inhibits the potential application of organoids in high-throughput drug screening. The second factor is the lack of vascularization, which can be defined as imperfections in the microenvironmental components. The majority of PDOs are devoid of stromal cells. As a consequence, they are incapable of reconstituting the microenvironment, including fibroblasts, endothelial cells, immune cells, and the ECM, and are devoid of signals that would otherwise promote organogenesis. This hampers the ability to accurately predict disease progression and enhancement. In order to mimic cancer-stromal cell interactions *in vivo*, 3D co-cultures, decellularization techniques, and microfluidic devices have been developed (Zhang et al., 2022). Future research place greater emphasis on utilizing patient-specific mesenchymal stromal cells *in vivo* to study the development of tumor-like organs. Furthermore, tumor-like organs are frequently constrained in size due to the absence of a vascular system, which impedes nutrient uptake. This limitation has been addressed by the advent of bioprinting and organ-on-a-chip technologies. With regard to drug development, organoids have been extensively investigated for their high-throughput screening properties, particularly in the identification of drug targets and biomarkers. They are also being employed as an emerging method to study anticancer drug sensitivity and resistance, as well as the development of combinatorial therapies. This provides the possibility of personalized and precise treatment for patients. It is noteworthy that in combination therapy, there is a need to place greater emphasis on the reuse of existing chemotherapy and targeted drugs. Drug repurposing, as an alternative to new drug development, offers the advantage of greater cost-effectiveness and time-saving. Srimongkol et al. constructed retinoblastoma (RB) organoids that recapitulate the genomic features of the original tumors (Srimongkol et al., 2023). Subsequently, 133 FDA-approved pharmaceutical agents were evaluated in RB organoids, with candidates selected based on potency and cytotoxicity. Sunitinib was identified as a more potent inhibitor of tumor cell proliferation in RB-like organs and a less toxic agent to normal retinal-like organs than melphalan or topotecan. These findings indicate that sunitinib may be a suitable candidate for repurposing in RB chemotherapy. In terms of precision therapy for cancer, the experimental basis is the construction of patient-derived organoids (PODs), which can preserve the cellular composition and genetic characteristics of individual patients. PODs can be used not only to screen patients for abnormalities and select individualized treatment regimens, but also to predict the patient’s response to chemotherapy and targeted therapies. Furthermore, organoid technology can be employed to predict a patient’s response to chemotherapy and targeted therapies. In the study conducted by Vlachogiannis et al. (2018), patient-derived organoid organ models were utilized to simulate treatment response in metastatic gastrointestinal cancer, thereby providing a powerful tool for personalized medicine. With the continuous advancement and optimization of this technology, it is reasonable to believe that organoid technology will play an even more important role in future biomedical research and clinical treatment.

Nevertheless, fundamental research on organoids is still confronted with a number of previous obstacles before genuinely and extensively employed in cancer therapy. Firstly, the majority of organoids are derived from epithelial cancers, and there is a paucity of knowledge regarding tumors of other tissue types. However, glioblastoma organoids have been reported (Wen et al., 2023). Secondly, the efficiency and purity of organoid culture must be enhanced, which is essential for high-throughput screening of drugs and precision therapy. Thirdly, the co-cultivation of organoids and microorganisms requires further investigation when examining the relationship between tumors and pathogens. Exploration has also been conducted, combining intestinal organoid culture with peripheral microinjection techniques (Puschhof et al., 2021). Finally, organoid research also raises additional ethical issues that require revisiting and potentially recalibrating ethical and legal policies (Bredenoord et al., 2017).

In the future, research on cancer-derived organoids will be deepened in a number of areas, including model construction, drug response, and precision therapy. Additionally, research will be conducted into emerging technologies designed to enhance the utility of organoids and the integration of organoids with other ‘omics’ data (Ha et al., 2023). This data can be used in systems biology to improve the ability to analyze the results of experiments in a systematic way. Furthermore, the utilization of organoid technologies in regenerative medicine has demonstrated considerable promise. For instance, transplantation experiments of cholangiocyte organoids have demonstrated that these organoids are capable of adapting to local environmental cues and restoring the expression of region-specific markers *in vitro* and following transplantation (Li H. et al., 2024). This has considerable implications for the regeneration of normal tissues and organs with a view to restoring function and improving prognosis following the resection of certain tumors. Our research aims to transform organoids into a basic research model and a therapy strategy for liver cancer. We will explore the potential of liver cancer organoids in understanding disease mechanisms and developing new treatments. Challenges remain in establishing robust models, maintaining the genetic and phenotypic stability of liver cancer organoids during long-term culture, and accurately recapitulating the tumor microenvironment. These challenges need to be addressed through technological innovations and interdisciplinary collaborations to fully realize the potential of organoids in liver cancer research and therapy. These findings not only advance our understanding of the biological properties of organoids, but also provide a scientific basis for future organoid-based therapeutic strategies.
