# Correction: Organoids in cancer therapies: a comprehensive review

**DOI:** 10.3389/fbioe.2025.1671862

**Published:** 2025-08-12

**Authors:** Jiang Xin-Yi, Wang Yan-Ran, Di Pin-Ru, Qian Shi-Yi, Jiang Hai-Tao

**Affiliations:** ^1^ Department of General Surgery, Ningbo No. 2 Hospital, Ningbo, Zhejiang, China; ^2^ School of Clinical Medicine, Hangzhou Medical College, Hangzhou, Zhejiang, China

**Keywords:** organoids, cancer therapy, disease modeling, drug screening, personalized medicine

In the published article, there was a mistake in the **author affiliations**. All authors should be attributed to both affiliations 1 and 2. Additionally, the affiliations were erroneously written in the wrong order.

The correct authors and their corresponding affiliations should be as follows:

“Jiang Xin-Yi^1,2^, Wang Yan-Ran^1,2^, Di Pin-Ru^1,2^, Qian Shi-Yi^1,2^, and Jiang Hai-Tao^1,2,^*”

“1Department of General Surgery, Ningbo No. 2 Hospital, Ningbo, Zhejiang, China”

“2School of Clinical Medicine, Hangzhou Medical College, Hangzhou, Zhejiang, China”

The original article has been updated.

